# Low-intensity focused ultrasound targeted microbubble destruction reduces tumor blood supply and sensitizes anti-PD-L1 immunotherapy

**DOI:** 10.3389/fbioe.2023.1173381

**Published:** 2023-04-17

**Authors:** Nianhong Wu, Yuting Cao, Ying Liu, Ying Zhou, Hongye He, Rui Tang, Li Wan, Can Wang, Xialin Xiong, Linhong Zhong, Pan Li

**Affiliations:** ^1^ Chongqing Key Laboratory of Ultrasound Molecular Imaging, Institute of Ultrasound Imaging and Department of Ultrasound, The Second Affiliated Hospital of Chongqing Medical University, Chongqing, China; ^2^ Department of Ultrasound, The Third People’s Hospital of Chengdu City, Chengdu, China

**Keywords:** immune checkpoint blockade, immune infiltration, antivascular therapy, cavitation, microbubble, low-intensity focused ultrasound

## Abstract

Immune checkpoint blockade (ICB) typified by anti-PD-1/PD-L1 antibodies as a revolutionary treatment for solid malignancies has been limited to a subset of patients due to poor immunogenicity and inadequate T cell infiltration. Unfortunately, no effective strategies combined with ICB therapy are available to overcome low therapeutic efficiency and severe side effects. Ultrasound-targeted microbubble destruction (UTMD) is an effective and safe technique holding the promise to decrease tumor blood perfusion and activate anti-tumor immune response based on the cavitation effect. Herein, we demonstrated a novel combinatorial therapeutic modality combining low-intensity focused ultrasound-targeted microbubble destruction (LIFU-TMD) with PD-L1 blockade. LIFU-TMD caused the rupture of abnormal blood vessels to deplete tumor blood perfusion and induced the tumor microenvironment (TME) transformation to sensitize anti-PD-L1 immunotherapy, which markedly inhibited 4T1 breast cancer’s growth in mice. We discovered immunogenic cell death (ICD) in a portion of cells induced by the cavitation effect from LIFU-TMD, characterized by the increased expression of calreticulin (CRT) on the tumor cell surface. Additionally, flow cytometry revealed substantially higher levels of dendritic cells (DCs) and CD8^+^ T cells in draining lymph nodes and tumor tissue, as induced by pro-inflammatory molecules like IL-12 and TNF-α. These suggest that LIFU-TMD as a simple, effective, and safe treatment option provides a clinically translatable strategy for enhancing ICB therapy.

## 1 Introduction

Tumor immunotherapy aims to kill tumor cells by activating and promoting autoimmune function to effectively eliminate tumors and improve prognosis, including immune checkpoint blockade (ICB), which has revolutionized the treatment strategy of malignant tumors (Zhang and Zhang, 2020; Li et al., 2021; Yi et al., 2022). Immune checkpoints, molecules of coinhibitory signaling pathways that maintain a normal immune response, are often utilized by cancer cells resulting in immune resistance (Zhang and Zhang, 2020). As one of the most widely used monoclonal antibodies for ICB, anti-PD-1/PD-L1 antibodies are designed to redirect T cells to cancer cells by blocking the binding of immune checkpoint PD-1 receptor on T cells and its ligand PD-L1 on tumor cells, thereby overcoming tumor immune evasion (Gambichler et al., 2020; Yamaguchi et al., 2022). Exciting therapeutic efficacy has been achieved in melanoma, lung cancer, lymphoma, and others (Song et al., 2020; Reck et al., 2021; Luke et al., 2022). Nevertheless, the overall response rate of checkpoint inhibition strategies is limited to 20%-30% in clinical practice because most “immune-cold” solid tumors are not responsive, which are characterized by the lack of intratumoral T cell infiltration or signals to stimulate T cell activation (Li et al., 2018; Bagchi et al., 2021; Zhang et al., 2022). Especially, it even suffers an extremely low objective response rate of about 5% in triple-negative breast cancer (Adams et al., 2019). For this reason, researchers have been exploring various combination therapies to modify the tumor microenvironment (TME) to turn ‘cold’ into “hot” tumors (Yadav et al., 2022). For instance, chemotherapy, radiotherapy, or photodynamic therapy can promote the expression of tumor antigens or PD-L1 on the cancer cell surface to enhance the tumor immune responses to anti-PD-1/PD-L1 antibodies (Krombach et al., 2019; Du et al., 2020; Su et al., 2021). Unfortunately, chemotherapy is based on the systemic administration of drugs that attack normal and tumor cells indiscriminately, resulting in serious adverse effects including gastrointestinal toxicity, neurotoxicity, myelosuppression and so on (Oun et al., 2018). Moreover, the induction of infiltration and accumulation of regulatory T cells (Tregs) and myeloid-derived suppressor cells (MDSCs) by radiotherapy lead to immunosuppression (Liang et al., 2017; De Martino et al., 2021). Photodynamic therapy has always struggled to overcome the limitations of low penetration depth, low target specificity, and premature leakage (Liu et al., 2020; Deng et al., 2021). Therefore, the search for new effective and safe combination therapies remains an essential and challenging task.

It is increasingly clear that the effectiveness of immunotherapy depends on the infiltration of immune effector cells (Morad et al., 2021). Ultrasound-targeted microbubble destruction (UTMD), a newly developed technique, can destroy the established aberrant tumor blood vessels and impede tumor angiogenesis by utilizing microjets, shock waves, and free radicals generated by ultrasonic cavitation (Wang et al., 2015; Han et al., 2022). More importantly, a range of potentially immunotherapeutic-related effects can be stimulated through ultrasonic thermal or cavitation mechanisms, including dendritic cells (DCs), effector T cells, and tumor-associated macrophages (Yin et al., 2021; Han et al., 2022). It has been reported that the combination of low-frequency ultrasound and microbubbles (MBs) can potentiate DCs differentiation and further induce T lymphocyte-mediated immune responses by downregulation of vascular endothelial growth factor in a mouse prostate cancer model (Zhang et al., 2017), the resulting acoustic cavitation can alter tumor-associated macrophages polarization and promote T cell infiltration (Abe et al., 2022). Therefore, UTMD demonstrates the great potential to convert ‘cold’ into ‘hot’ tumors through multiple immunomodulating mechanisms, thereby sensitizing cancer immunotherapy. Notably, low-intensity ultrasound combined with MBs holds undeniable advantages of efficiency and safety, precise targeting, clinical availability, and high penetration (Arulpragasam et al., 2022), which enables it to be an ideal option to enhance ICB therapy.

**FIGURE 1:**
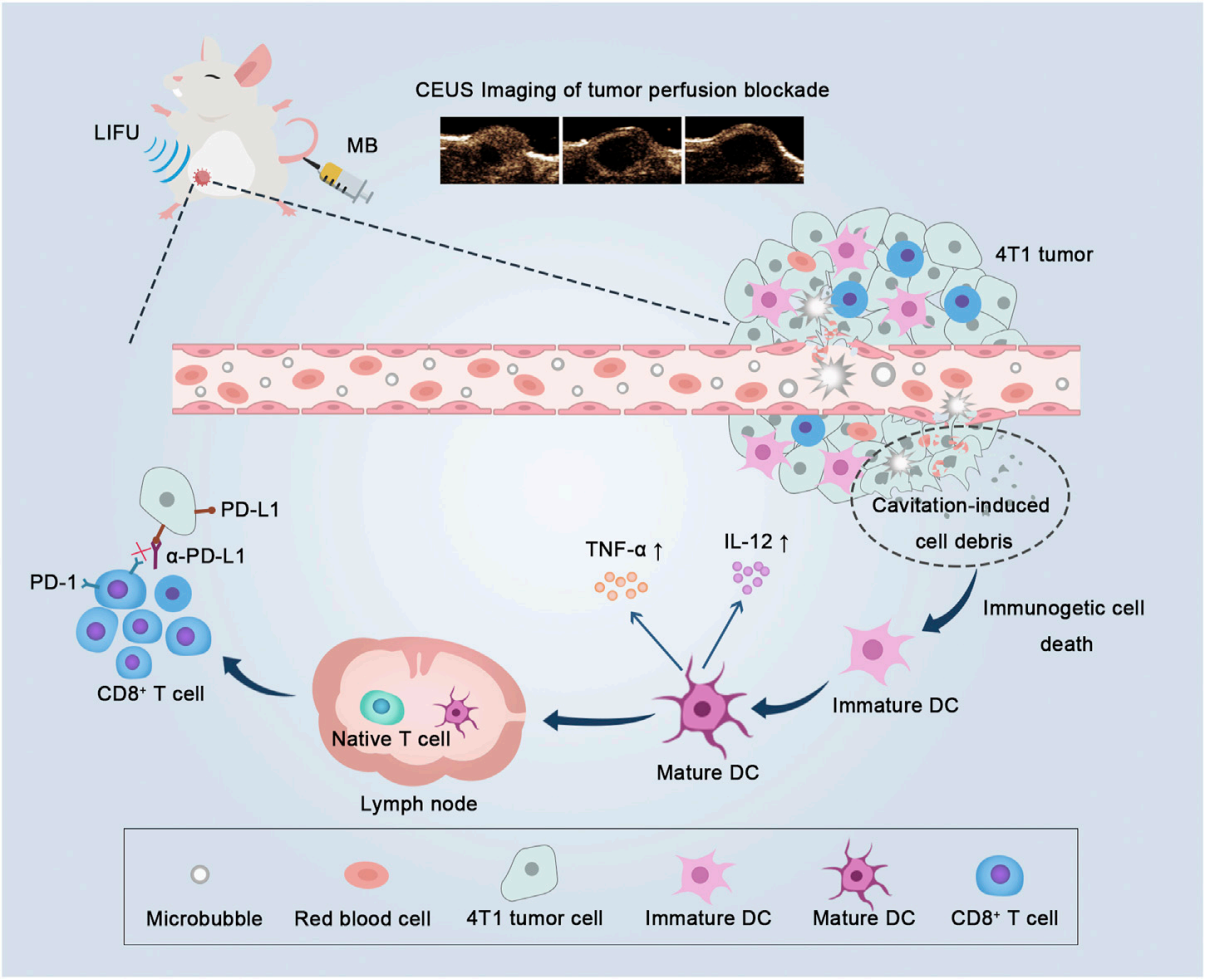
Low-intensity focused ultrasound-targeted microbubble destruction (LIFU-TMD) damages tumor vascular endothelial cells and starves tumor cells by cutting off the blood supply, and simultaneously tumor cell debris induced by the cavitation effect further stimulates dendritic cells (DCs) maturation, and ultimately activating CD8^+^ T cells, thereby enhancing the efficacy of PD-L1 blockade.

To date, the anti-tumor effects and synergistic mechanisms remain to be explored in the combination of ultrasound-induced biological effects and immunotherapeutic approaches. In this study, we investigated the anti-tumor effect of low-intensity focused ultrasound-targeted microbubble destruction (LIFU-TMD) treatment combined with ICB therapy and explored the potential mechanisms in a 4T1 orthotopic breast cancer model in mice. We found that LIFU-TMD treatment significantly reduced tumor growth by mechanically destroying tumor vascular endothelium, which led to a substantial decrease in tumor blood perfusion. Moreover, the ultrasonic cavitation-mediated damage-induced immunogenic cell death (ICD), improved the efficiency of anti-PD-L1 treatment by stimulating DCs maturation and enhancing CD8^+^ T cells infiltration in tumor tissue (Figure 1). Considering the accessibility of low-intensity ultrasound and the superb safety of MB contrast agents (Sirsi and Borden, 2009), the combination of LIFU-TMD and anti-PD-L1 immunotherapy holds great promise for clinical translation.

## 2 Materials and methods

### 2.1 Cell lines and animals

4T1 murine breast carcinoma cell line was provided by Chongqing Medical University. 4T1 cells were cultured in RPMI 1640 medium (Gibco) with 10% fetal bovine serum (Biological Industries) and 1% penicillin/streptomycin (Gibco) as dietary supplements at 37°C and 5% CO_2_. Female Balb/c mice at the age of 6 to 8 weeks old were bought from the Chongqing Medical University Animal Center. The mice were housed in specified pathogen-free environments with a 12-h light/dark cycle, providing food and water *ad libitum*. The *in vivo* experimental protocols were approved by the Ethics Committee of the Second Affiliated Hospital of Chongqing Medical University (Ethical approval number: (2022) 103).

### 2.2 MB preparation and characterization

1,2-dipalmitoyl-sn-glycero-3-phosphocholine (DPPC), 1,2-Distearoyl-sn-glycero-3-phosphoethanolamine-N-[methoxy (polyethylene glycol)-2000] (DSPE-PEG2000) were purchased from Avanti Polar Lipids Inc. and Xi’an Ruixi Biological Technology Co., respectively. DPPC (10 mg) and DSPE-PEG2000 (4 mg) were dissolved in 1 mL of glycerol-phosphate-buffered saline (PBS) suspension to make a lipid film solution by a thin-film hydration method and stored in a 2 mL vial. Perfluoropropane was then poured into the vial. Finally, MBs were obtained by shaking for 40 s using the dental amalgamators (Shanghai Medical Instruments Co., Ltd.). The morphology of MB was observed by an optical microscope (Olympus). The particle size and zeta potential were measured using dynamic light scattering (Malvern).

### 2.3 *In vivo* anticancer treatment

4T1 cells in the logarithmic growth phase were collected and resuspended in PBS to a concentration of 5×10^7^ cells/mL. Subsequently, the 4T1 orthotopic breast cancer model was established by injection with 100 μL 4T1 cell suspensions into the fifth right mammary fat pad. When tumors reached 50-100 mm^3^ (about 7 days after 4T1 tumor cell inoculation), for the anti-tumor treatment investigation, four groups of mice were tested (n = 5 per group): 1) Control (no treatment), 2) LIFU (no MB), 3) MB (no LIFU), and 4) MB + LIFU. A self-developed LIFU instrument (Chongqing Key Laboratory of Ultrasound Molecular Imaging) was used in this study, which consisted of a hand-held transducer with a diameter of 1 cm, a focal length of 1.5 cm, a focus area of 0.4 cm^2^, driving frequency of 1.0 MHz, acoustic intensity controllable by the user at the focal spot of 0.5-3.5 W/cm^2^ (continuous-wave mode) or 1.0-8.0 W/cm^2^ (pulsed-wave mode), and a duty cycle of 50%. The acoustic intensity was measured with a hydrophone (HNA-0400, ONDA Corporation, California, CA, United States). The parameters of ultrasound are 1.0 MHz, 3 W/cm^2^, and 50% duty cycle with 5 min sonication duration. 4T1 tumor-bearing mice were treated with LIFU immediately after intravenous injection of MB (200 μL suspension) on day 0, 1, 2, 3 and 4. During the treatment, the ultrasonic probe was moved around the tumor so that the whole tumor can be fully covered. The tumor volume was monitored every second day with the following formula: $Volume=\left(Length\times {Width}^{2}\right)/2$ mm^3^. The mouse weight was recorded every other day until 11 days after treatment. Three tumor-bearing mouse from each group was selected randomly and euthanized by cervical dislocation 3 days after the final treatment. The tumors were then collected and fixed with 4% paraformaldehyde for H&E staining, proliferating cell nuclear antigen (PCNA), and terminal deoxynucleotidyl transferase dUTP nick end labeling (TUNEL) immunofluorescence analysis.

### 2.4 Antivascular effect evaluation

To explore the antivascular effect of LIFU-TMD treatment, the tumor perfusion was evaluated using an ultrasonic scanner from the Vevo^®^ 2100 Imaging System (VisualSonics Inc.) with the LZ-250 sensor in dual contrast mode (frequency: 18 MHz, power: 4%, contrast gain: 40.0 dB, and 2D gain: 18.0 dB), and the focal point was adjusted to the middle or the lower edge of the lesion. The mice were injected with 200 μL of MB suspension via the tail vein and treated with the same as the above acoustic parameters. B-mode and contrast-enhanced ultrasound (CEUS) imaging were performed before treatment (Pre-treatment), immediately after treatment (Post-0 h), and 24 h later (Post-24 h) with another 200 μL of MB injection. A time-intensity curve of the contrast signals in the region of interest was analyzed using QontraXt software (Amid, Milan, Italy), and three-dimensional pseudo-color images were generated based on the computer simulation of the acoustic intensity distribution in the tumors at the peak time point, and the mean peak intensity (PI) value of tumor perfusion was recorded and compared to evaluate the antivascular effect induced by LIFU-TMD.

Next, mice with 4T1 tumors were sacrificed. The histological changes were checked by H&E staining, and the tumor vessels were marked by CD31 antibody staining to assess microvessel density (MVD). MVD was counted according to the following criteria: The highest vessel density areas were selected at ×20 magnification, and then three fields were selected and counted under ×40 magnification.

### 2.5 Immune cells infiltration

To observe the effect of LIFU-TMD treatment on TME, calreticulin (CRT) protein exposed on the cell surface as a distinct biomarker of ICD was investigated by immunofluorescence staining (Han et al., 2019). Moreover, the maturation of DCs is essential for the activation of T lymphocytes to stimulate an efficient specific immune response (Marciscano and Anandasabapathy, 2021). Therefore, we evaluated changes in cytotoxic T lymphocytes (CTLs) within the tumor by flow cytometry. The following provides specific steps. Four groups of tumor-bearing mice were tested (n = 3 per group): 1) Control (no treatment), 2) LIFU (no MB), 3) MB (no LIFU), and 4) MB + LIFU. LIFU was performed immediately after intravenous injection of 200 μL MB suspension on day 0, 1, 2, 3 and 4. All the mice were sacrificed on day 7 after the last treatment, then the tumors and draining lymph nodes were collected and cut into fragments. Subsequently, enzyme digestion solution (0.025 mg/mL DNase I, 0.05 mg/mL Hyaluronidase, and 0.1 mg/mL collagenase IV) was added and the resulting suspension was incubated at 37°C for 40 min to produce a single-cell suspension. After the depletion of the red blood cells (RBCs) with red blood cell lysis buffer (Biosharp), the single-cell suspension was washed with PBS and stained with fluorescently labeled antibodies according to the protocol recommended by the manufacturer. Finally, the prepared cell suspension was analyzed by flow cytometry. The following antibodies were purchased from BioLegend: FITC anti-mouse CD3 (100204), PE anti-mouse CD4 (100408), APC anti-mouse CD8a (100712), FITC anti-mouse CD11c (117306), PE anti-mouse CD80 (104708), APC anti-mouse CD86 (105012), and Alexa Fluor^®^ 647 anti-mouse FOXP3 (126408). At the same time, TNF-α and IL-12 in serum from different groups of mice were tested by ELISA kits (Jiangsu Meimian Industrial Co.). In forward scatter (FSC)/side scatter (SSC) plots of flow cytometry, lymphocyte population size and granularity are analyzed. As compensation controls, we performed single-dye stains. The expression of CD11c^+^ lymphocytes was detected. Finally, Matured DCs were defined as the CD80^+^ CD86^+^ subpopulation of the CD11c^+^ subset. Similarly, CD4^+^ and CD8^+^ T cells were subdivided from the CD3^+^ T lymphocyte subset within the lymphocyte size/structure gate, and CD3^+^ CD8^+^ T lymphocytes were defined as CTLs. Tregs were identified by separating CD4^+^ Foxp3^+^ cells from the CD3^+^ subpopulation.

### 2.6 *In vivo* synergistic therapy

For the synergistic therapy experiment, four groups of orthotopic 4T1-bearing mice on the right were tested (n = 5 per group): 1) Control (no treatment), 2) PD-L1, 3) MB + LIFU, and 4) MB + LIFU + PD-L1. On day 0, 1, 2, 3 and 4, MB suspension was intravenously injected followed by LIFU (1.0 MHz, 3 W/cm^2^, 50% duty cycle, and 5 min). Anti-PD-L1 antibody (1.5 mg/kg) was intravenously injected on day 1, 4 and 7. The mouse weight and volume were monitored every other day. Three mice were randomly sacrificed on day 11 after treatment and the obtained tumor tissue was immunofluorescently labeled with CD8 antibody.

### 2.7 Statistical analysis

Data for statistical analysis were expressed as means ± standard deviations, One-way analysis of variance (ANOVA), followed by Tukey correction, was used to make multiple comparisons. The changes in MVD were examined by Student’s two-tailed *t*-test. *p < 0.05, **p < 0.01, ***p < 0.001, ****p < 0.0001.

## 3 Results

### 3.1 LIFU-TMD treatment inhibits tumor growth

**FIGURE 2:**
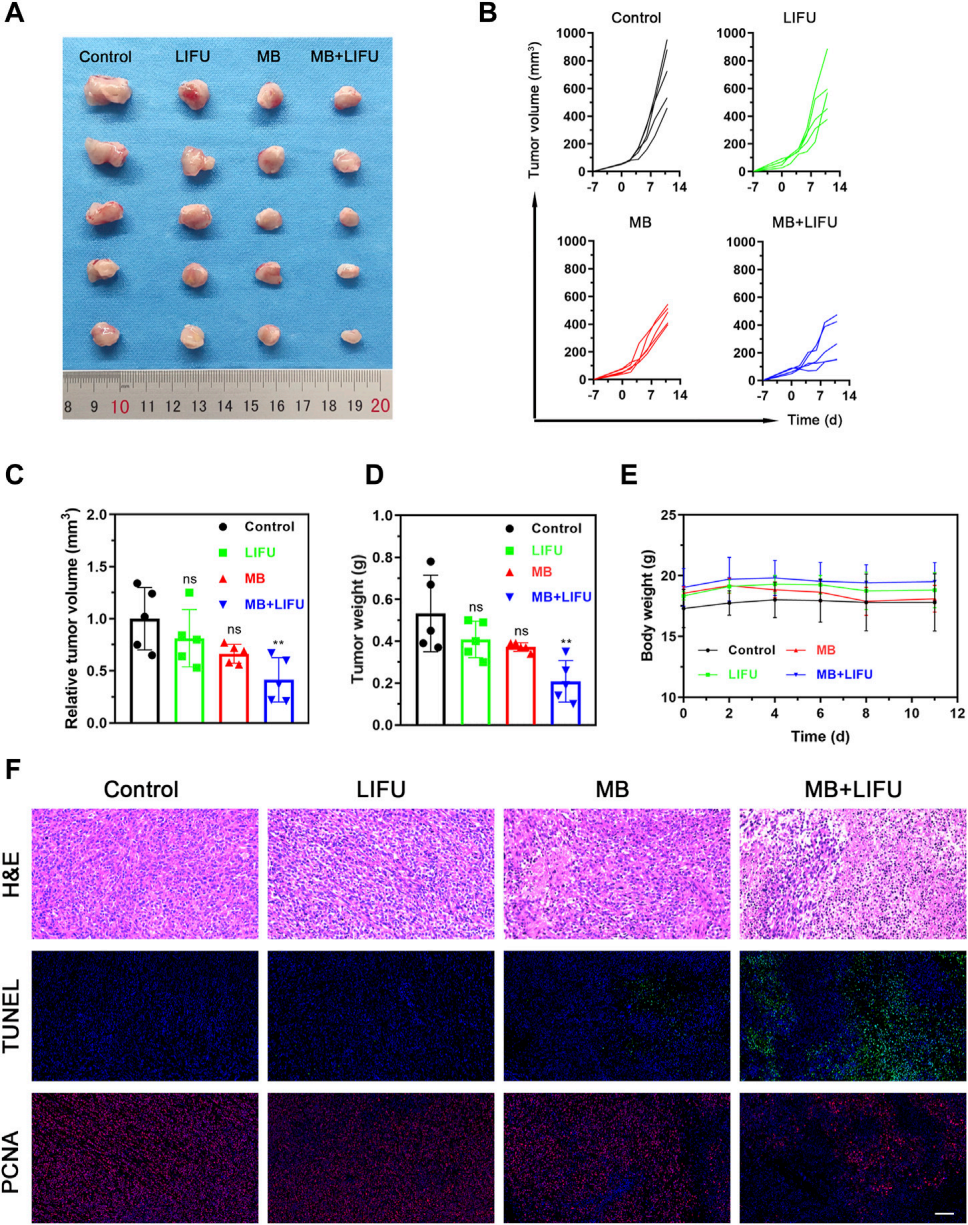
Evaluations on the LIFU-TMD treatment anti-tumor effectiveness *in vivo*. **(A)** Representative tumor images of the 4T1 tumors of diverse groups on day 11 after different treatments. Individual tumor growth curves **(B)** and the relative tumor volume **(C)** of the 4T1 tumors. **(D)** Tumor weights of the 4T1 tumors after different treatments. **(E)** Body weight change in each group. **(F)** H&E staining, TUNEL staining (green fluorescence) and PCNA staining images (red fluorescence) of the 4T1 tumors. Scale bar: 100 μm. Data are presented as mean ± SD (*n* = 5), *p < 0.05, **p < 0.01.

Following the effective creation of milky white microbubbles with a potential of −5.12 ± 0.99 mV and a particle size of 1957 ± 53.37 nm in a single-peaked distribution (Supplementary Figures S1–S5), we assessed the impact of LIFU-TMD therapy on tumor growth in a 4T1 *in situ* breast cancer model. The experimental design was displayed in Supplementary Figure S6. As shown in Figures 2A–D, treatment with MB or LIFU alone has no obvious anti-tumor effect, while MB + LIFU treatment significantly inhibited tumor growth. And LIFU-TMD treatment exhibited favorable safety in view of the negligible fluctuation in the body weight of mice (Figure 2E). To further confirm the therapeutic efficacy, tumors were collected and sectioned for H&E, TUNEL, and PCNA staining on the third day after treatment. Mass necrosis and apoptosis were found in the group with MB + LIFU treatment, and the green fluorescence signal of dead cells was notably strengthened, whereas cells with proliferation exhibited a weaker red signal, indicating LIFU-TMD therapy could promote potent cancer cell apoptosis and inhibit tumor cell proliferation (Figure 2F). These results encouraged us to further explore the underlying mechanisms of LIFU-TMD treatment.

### 3.2 LIFU-TMD treatment blocks tumor blood perfusion and causes histological damage

**FIGURE 3:**
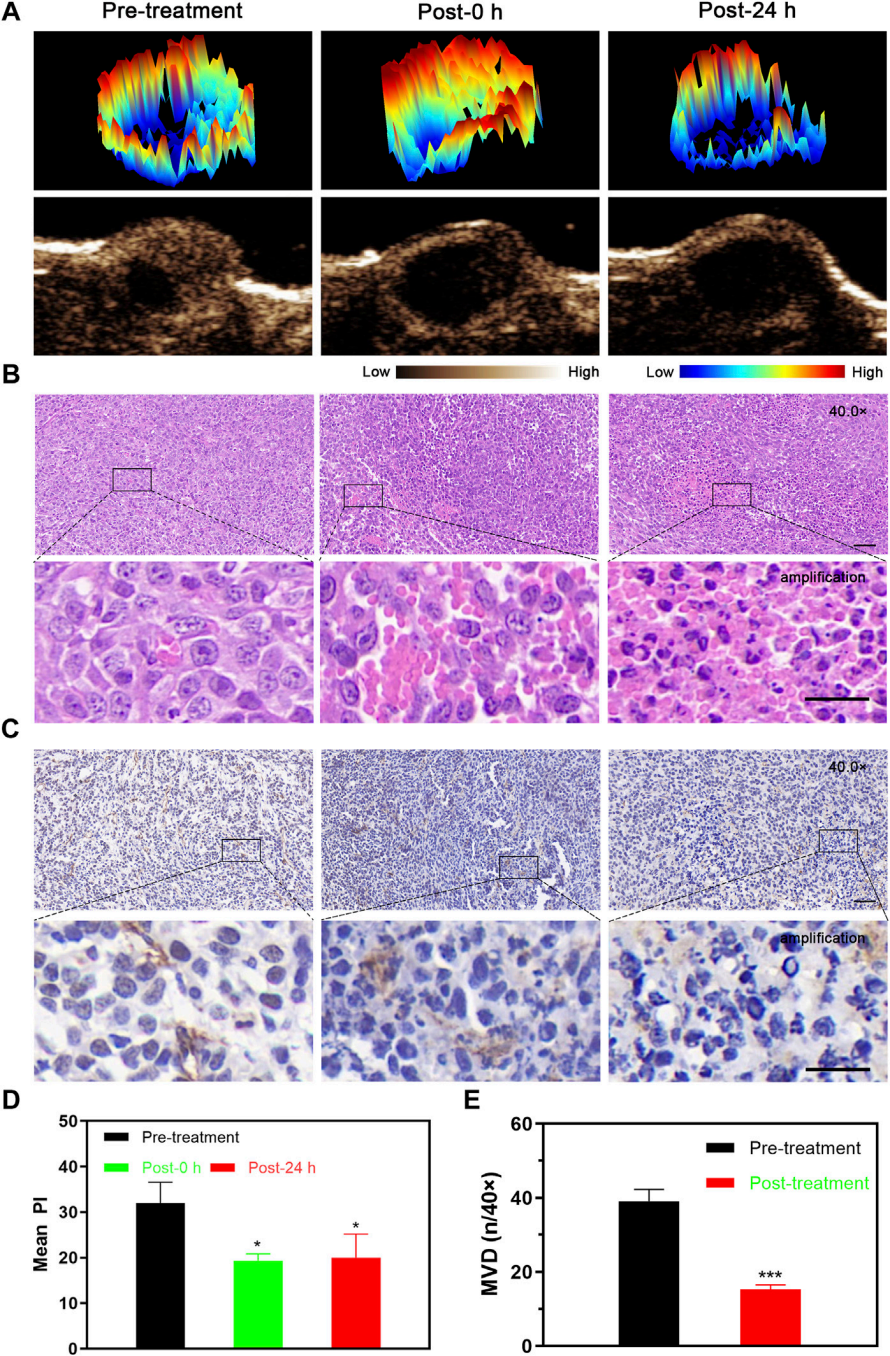
Tumor perfusion and histological changes of microvessels at different time points after LIFU-TMD therapy. **(A)** Representative contrast-enhanced ultrasound (CEUS) and three-dimensional pseudo-color images of the 4T1 tumor. H&E **(B)** and immunohistochemical staining for the endothelial marker CD31 with brown color **(C)** in the 4T1 tumor by light microscopy at before treatment (Pre-treatment), immediately after treatment (Post-0 h) and 24 h later (Post-24 h); scale bar: 50 μm for 40.0x images, 20 μm for amplification. **(D)** Mean peak intensity (PI) of tumors at 3-time points. **(E)** Quantitative analysis of the microvessel density (MVD) at different times. Data are presented as mean ± SD (*n* = 3), *p < 0.05, **p < 0.01, ***p < 0.001.

After LIFU-TMD treatment, the tumor blood perfusion significantly decreased and was not substantially recovered 24 h later (Figure 3A; Supplementary Figure S7), and the mean PI values showed a similar trend (Figure 3D).

H&E staining results verified the disrupted microvessels in tumor tissue after treatment, the tubular structure disappeared and RBCs extravagated into the interstitial space. Flaky necrosis with pyknosis, karyorrhexis, and karyolysis was shown in tumor tissue 24 h later (Figure 3B). Next, CD31 immunohistochemistry staining results revealed that the vascular morphology of the tumor was intact and clearly visible before LIFU-TMD treatment, while LIFU-TMD mediated damage to tumor vascular endothelium and structure was confirmed by the unevenly staining and invisible tubular architectures after LIFU irradiation (Figure 3C). MVD expression was markedly reduced at 24 h post-treatment (Figure 3E). Taking these findings together, LIFU-TMD treatment inhibited tumor growth mainly by blocking blood perfusion and causing tumor cell damage.

### 3.3 LIFU-TMD treatment enhances immune effector cells infiltration

**FIGURE 4:**
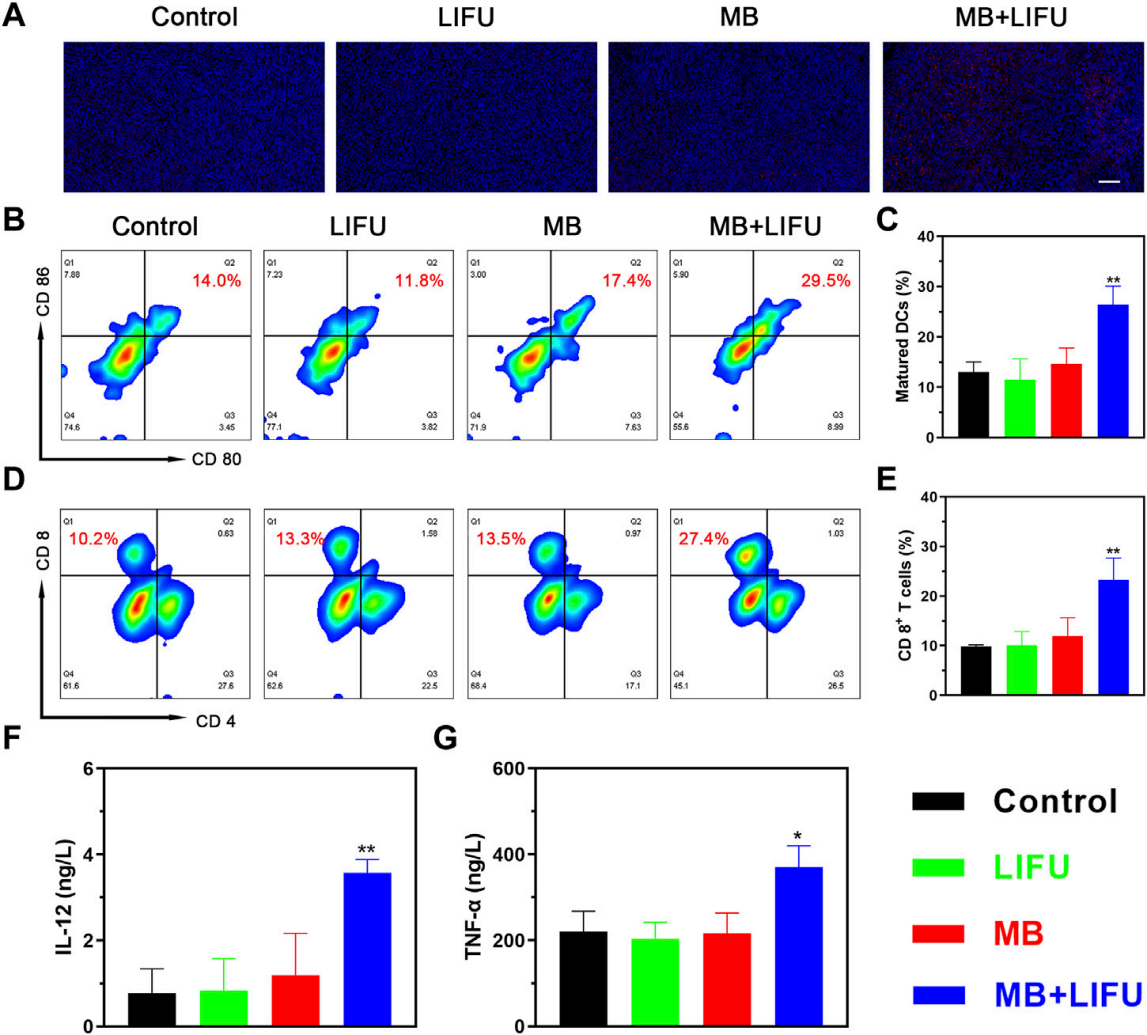
*In vivo* immunomodulating mechanisms. **(A)** Immunofluorescence staining of 4T1 tumors showing the induction of calreticulin (CRT) after different treatments (scale bar: 100 μm). **(B)** The matured DCs (CD11c^+^CD80^+^CD86^+^) in tumor-draining lymph nodes of mice after different treatments analyzed by flow cytometry and **(C)** the corresponding quantitative data. **(D)** Flow cytometric analysis of cytotoxic T lymphocytes (CTLs; CD3^+^CD8^+^) in 4T1 tumor-bearing mice and **(E)** the corresponding quantification of CTLs. **(F, G)** Cytokine levels in sera measured by ELISA assay. All data are presented as mean ± SD (*n* = 3), *p < 0.05, **p < 0.01.

Encouraged by the release of damage-associated molecular patterns (DAMPs) signals *in vitro*, including CRT, adenosine triphosphate (ATP), and high mobility group box protein B1 (HMGB1), whose results were reported in the Supplementary Figures S8–S10, the *in vivo* immune responses were evaluated next. As shown in Figure 4A, a higher expression of CRT with a stronger fluorescence signal was found in the MB + LIFU group, indicating enhanced ICD after LIFU-TMD treatment. And the matured DCs proportion in tumor-draining lymph nodes was upregulated as analyzed by flow cytometry, which was significantly higher in the MB + LIFU group (26.40% ± 3.70%) compared to that in the control group (13.03% ± 2.03%) (Figures 4B, C). In addition, we observed a significant increase of CTLs infiltration in the MB + LIFU group (23.27% ± 4.42%) compared to that in other groups (Figures 4D, E). Serum cytokines, including IL-12 and TNF-α, play an important role in recruiting immune cells. ELISA results demonstrated that IL-12 and TNF-α secretion levels increased in serum in the LIFU-TMD treatment group (Figure 4F, G), which corresponded with the findings of DCs maturation and CTLs infiltration. The percentage range for Tregs is slightly smaller than for groups Control and LIFU, whereas there was no statistical difference among all groups (Figure S11-12).

### 3.4 LIFU-TMD treatment increases anticancer responsiveness to anti-PD-L1 therapy

**FIGURE 5:**
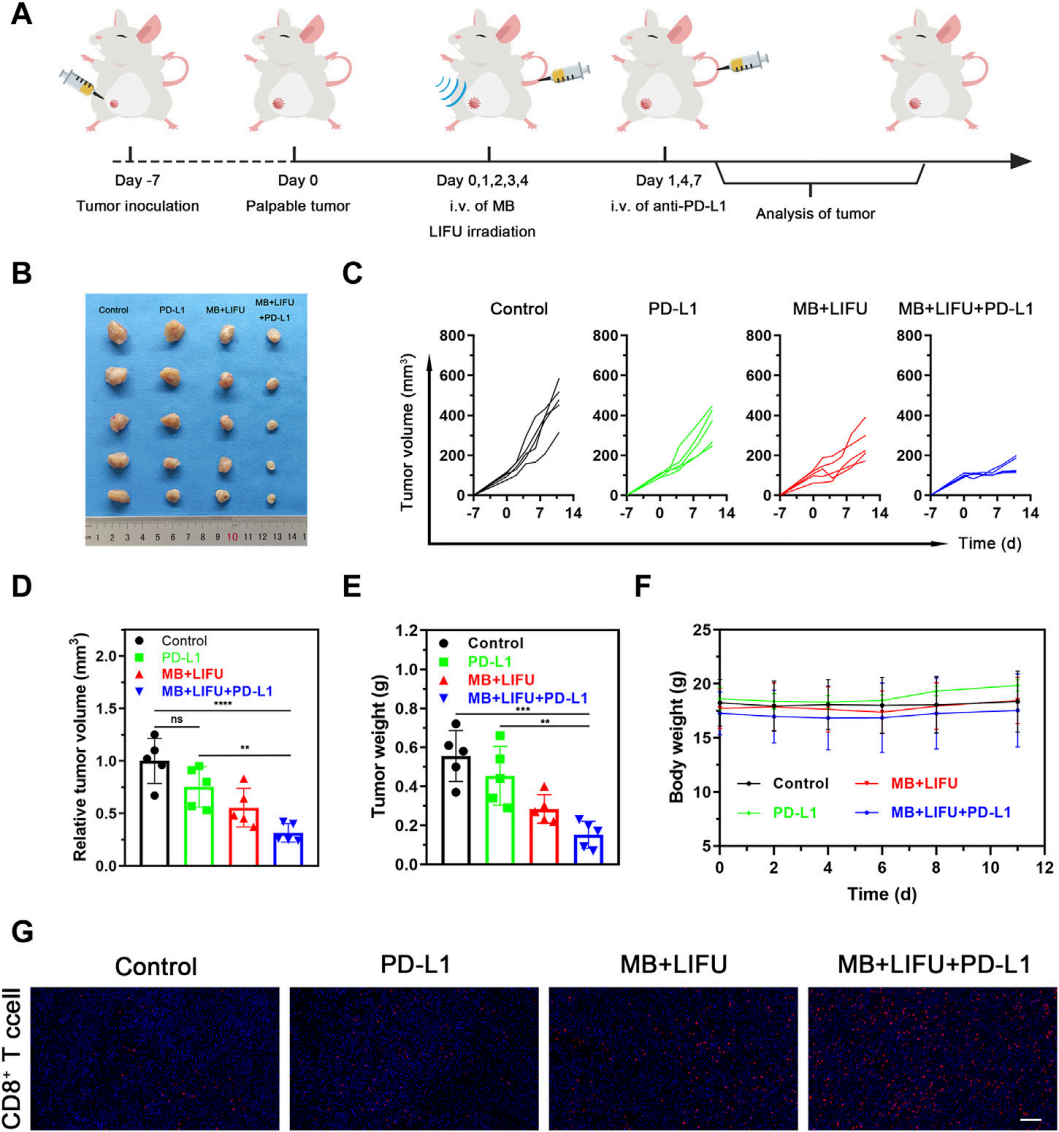
Evaluations of anti-tumor efficacy *in vivo* of LIFU-TMD treatment plus PD-L1 blockade. **(A)** Schematic illustration of LIFU-TMD treatment plus anti-PD-L1 antibodies to suppress the development of the tumors. (*n* = 5) **(B)** Representative tumor images of the 4T1 tumors of various groups after different treatments. Individual tumor growth curves **(C)**, the relative tumor volume **(D)** and tumor weights **(E)** of the 4T1 tumors after different treatments (*n* = 5). **(F)** Body weight change in each group (*n* = 5). **(G)** Immunofluorescence staining images of CD8^+^ T cell the 4T1 tumors. Scale bar: 100 μm. Data are presented as mean ± SD, *p < 0.05, **p < 0.01, ***p < 0.001, ****p < 0.0001.

The efficacy of anti-PD-L1 immunotherapy is limited by insufficient CTLs infiltration within the tumor. Encouraged by the verified satisfactory immunostimulation effect, we next evaluated the synergistic effectiveness of LIFU-TMD in combination with anti-PD-L1 therapy. The combinatory therapy regime was illustrated in Figure 5A. As shown in Figures 5B–E, tumor growth reduction was greatest in the MB + LIFU + PD-L1 group based on the results of tumor volume and weight. LIFU-TMD treatment could inhibit tumor growth as confirmed in the above experiment, while anti-PD-L1 immunotherapy showed no evident tumor inhibitory effect. No obvious fluctuations in the body weight of mice were found during the treatment (Figure 5F), indicating good therapeutic safety. Similarly, we observed a remarkable increase of activated CD8^+^ T cell infiltration in the MB + LIFU + PD-L1 group (Figure 5G). Therefore, LIFU-TMD treatment not only inhibits tumor growth but simultaneously activates an immune response to anti-PD-L1 antibodies leading to synergistic therapeutic efficacy.

### 3.5 Biosafety evaluation

**FIGURE 6:**
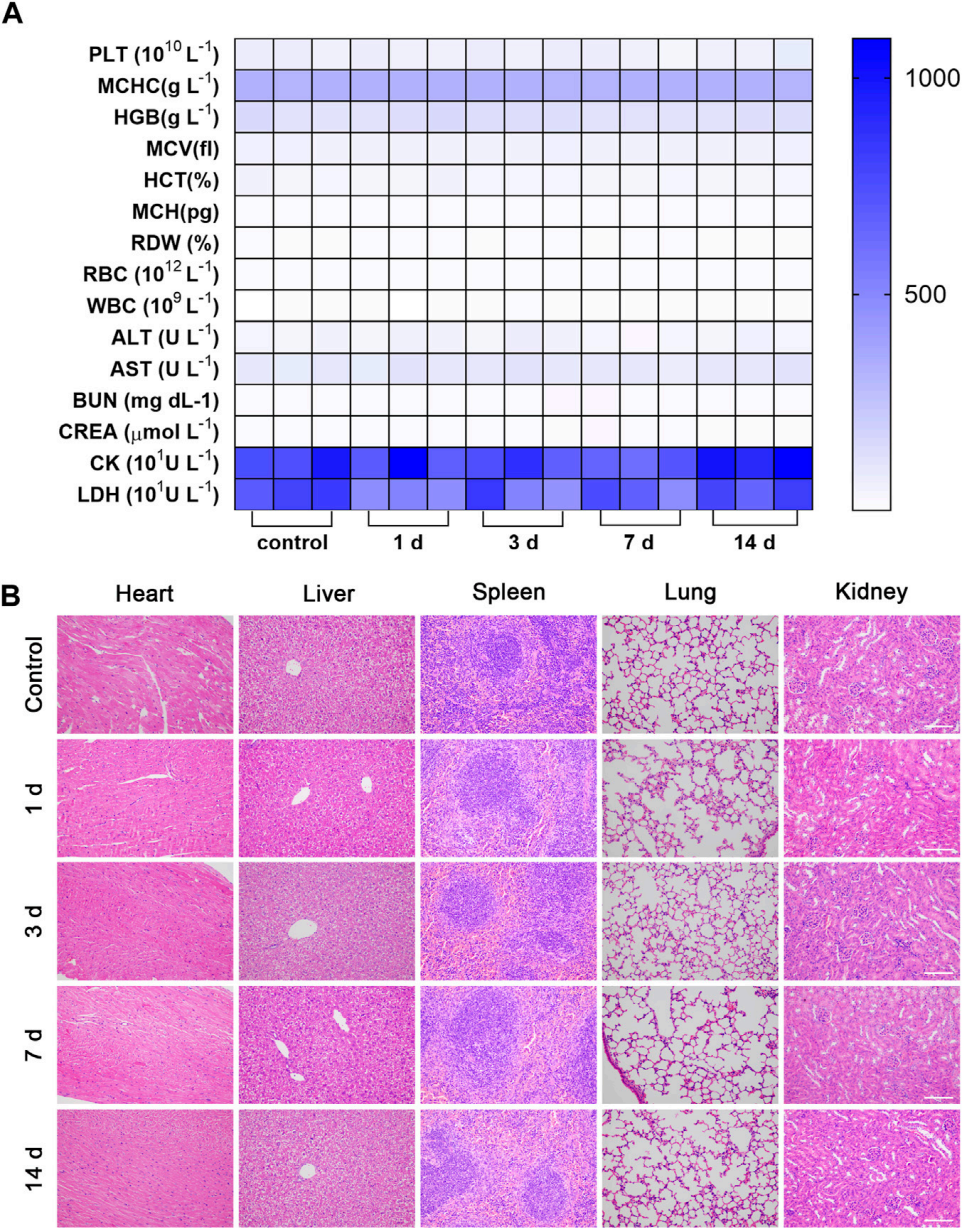
Biosafety evaluation. The hematology indicators **(A)** and H&E staining of major organs of **(B)** Balb/c mice 14 days after intravenous injection of MBs (*n* = 3, scale bar: 100 μm).

As shown in Supplementary Figures S13–S16, the MB and LIFU groups showed nearly no toxicity to the HUVECs and 4T1 cells. When the concentration of MBs increased, the viability of 4T1 cells reduced significantly after irradiation of LIFU (Supplementary Figure S17). Accordingly, LIFU or MB alone demonstrated good biocompatibility *in vitro*, while increasing the MBs concentration (≥20%) resulted in targeted killing of 4T1 tumor cells through LIFU. Consistently, the body weight was not affected by MBs injection in tail veins (Supplementary Figure S18). Furthermore, routine blood and serum biochemistry, as well as H&E staining of major organs, revealed no significant systematic toxicity, suggesting good biocompatibility of MBs *in vivo* (Figures 6A, B).

## 4 Discussion

Recently, there has been a lot of interest in the use of nanocarriers to modify the TME to improve the effectiveness of ICBs, including the use of low-intensity ultrasound stimulation nanoparticles loaded with acoustic sensitizers to trigger an anti-tumor immune response (Yue et al., 2019; Huang et al., 2021; Yadav et al., 2022). Nevertheless, it is difficult to apply nanomedicines in clinical practice due to unclear release mechanisms, uncertainty about potential toxicity, and incomplete pharmacokinetic behavior of drug-loaded nanoparticles *in vivo* (Karimi et al., 2016; Su et al., 2019). Therefore, in this study, we aimed to seek a novel strategy to improve patients’ response to ICBs to address unmet clinical needs. As a commonly used contrast enhancement agent clinically, MBs caught our attention with a high safety and stability profile. Our earlier studies exhibited no substantial alterations in key organs in mice treated with LIFU (Deng et al., 2021; Jiang et al., 2022). We selected LIFU-mediated MB destruction treatment to hypothesize synergy with the combined anti-PD-L1 antibodies. Fortunately, we demonstrated that LIFU-TMD treatment generated a similar anti-tumor effect, and the combination with anti-PD-L1 antibodies successfully inhibited the growth of cold tumors.

Since the aberrant vascular system due to the high rate of angiogenesis in TME is known to play a critical role in promoting tumor growth and metastasis, reduction in tumor perfusion is particularly attractive. The antivascular effect of ultrasound-mediated MB disruption has been adequately documented by several studies (Todorova et al., 2013; Wang et al., 2015; Shen et al., 2017; Wu et al., 2020; Yu et al., 2022), which is consistent with our work. Based on imaging and histological results, we found that tumor perfusion was visibly reduced after LIFU-TMD treatment and MVD was decreased 24 h later. As disrupting the established abnormal vasculature, the tumor cells would be directly starved without oxygen and nutrients from tumor blood vessels (Ho et al., 2017; Snipstad et al., 2021), in line with the results of sustained perfusion reduction caused by LIFU-TMD.

Notably, while the impact of ultrasound cavitation effect in regulating immune cell infiltration has been documented by pieces of evidence, the mechanisms concerning the adaptive immune response induced by low-intensity ultrasound are still partially explored (Hunt et al., 2015; Ho et al., 2020; Liu et al., 2022). A recent study reported that a cytotoxic T cell (T-cyt) dependent mechanism plausibly existed in antivascular ultrasound stimulation of MBs-induced anti-tumor immune response longitudinal growth studies by the use of the CT26 model (Bulner et al., 2019). Our results suggested that in addition to enhancing CTLs infiltration, LIFU-TMD treatment had a broader impact on the TME of 4T1 breast cancer, which has been reported to convert to immunosuppressive TME in a natural course over 11 days (Cohen et al., 2020). To activate the anti-tumor immune response, several critical steps must be taken. 1) Immature DCs can specifically recognize antigens based on their expressed molecules and are transformed into matured DCs upon stimulation by maturation signals. 2) T cells in lymph nodes are activated by matured DCs presenting antigens. 3) Activated T cells infiltrate tumor tissue and kill cancer cells (Somarribas Patterson and Vardhana, 2021; Bao and Xie, 2022). The preliminary data we collected in this study was consistent with the above principles: 1) DAMPs signals changed in conjunction with tumor fragmentation induced by the ultrasonic cavitation effect. CRT served as an ‘eat-me’ signal to stimulate the antigen-presenting function of DCs, while ATP functioned as a “find-me” signal to recruit DCs, and HMGB1 assisted in the maturation of DCs (Bao and Xie, 2022; Li et al., 2022). As expected, the percentage of matured DCs was approximately 1.6 times greater than that in the other groups. Also, the secretion levels of pro-inflammatory cytokines in serum, including IL-12 and TNF-α, were increased in the ELISA assay, which were able to recruit and activate DCs and enhance antigen-specific immunity (Karan, 2018). 2) The percentage of CD3^+^CD8^+^ T cells was nearly 2.5-fold higher compared to that in the control group, confirming the activation and infiltration of effector T cells.

Currently, the lack of CTLs infiltration in solid tumors limits patients’ response to ICB therapy. Several previous studies implied that one of the immune-supportive properties of low-intensity ultrasound-mediated treatment depended on the development of inflamed TME supported by CD8^+^ T cells, and its establishment was proved in this study. In addition, drug distribution in the TME can be affected by abnormal vasculature and high interstitial fluid pressure (IFP). It has been confirmed that vascular disrupting agents (VDA) can effectively reduce high IFP and improve tumor vascular permeability by influencing tumor blood flow (Bouzin and Feron, 2007; Liu et al., 2022). Due to intravenous administration, VDA would cause side effects by affecting some damaged normal blood vessels in the systemic circulation. Attractively, UTMD can destroy tumor blood vessels in the site through cavitation to reduce adverse effects. And vascular rupture generated by disruption of micro- or nano-droplets with ultrasound can reduce IFP, break the pore size limitation between endothelial cells, and expand tissue space to facilitate drug penetration (Ho and Yeh, 2017; Cao et al., 2018; Ho et al., 2018; Xi et al., 2022; Zheng et al., 2023). In light of the above theory, it is reasonable to speculate that the destruction of tumor vasculature by LIFU-TMD treatment caused incomplete vessel wall structure and decreased IFP, which could promote the penetration of anti-PD-L1 antibodies into tumors through the enlarged vascular space. Therefore, LIFU-TMD potentiated the efficacy of PD-L1 blockade immunotherapy through multiple mechanisms in our study.

There are a few limitations to our research. Firstly, the investigation of the immune cell infiltration for a broader range of time points (such as 24 h, 3 days and 5 days post-treatment) is warranted, which would be more beneficial for us to determine the optimal time of administration of anti-PD-L1. Secondly, the work validated the short-term anti-tumor effects of LIFU-TMD, while it will be desirable to also look into the long-term impacts of tumor growth inhibition.

Overall, in this study, we observed that LIFU-mediated MB destruction led to the rupture of tumor microvasculatures causing a substantial decrease of tumor perfusion and directly resulting in massive tumor cell death. Notably, we found that a portion of tumor cells underwent ICD accompanied by the release of DAMPs, which further stimulated DC maturation and recruitment of T cells into the TME. These findings lay the foundation for the further combination of LIFU-TMD treatment with PD-L1 blockade, which resulted in synergistic anti-tumor efficacy. It is well known that MBs possess good biosafety with broad clinical application and LIFU is endowed with safety, non-invasiveness, accessibility, and simple operation, we thereby can expect the combinatory therapy of LIFU-TMD with PD-L1 blockade will be a promising strategy for clinical translation.

## Data Availability

The data presented in this study are available from the corresponding author on reasonable request.
