# Supplementary material for: Low-intensity focused ultrasound targeted microbubble destruction reduces tumor blood supply and sensitizes anti-PD-L1 immunotherapy
Source: Front Bioeng Biotechnol. 2023 Apr 17;11:1173381. doi: 10.3389/fbioe.2023.1173381 (PMC10150078; doi:10.3389/fbioe.2023.1173381)
Supplement: Supplementary file 1 [file DataSheet1.docx]

Supplementary Material

Low-intensity focused ultrasound targeted microbubble destruction reduces tumor blood supply and sensitizes anti-PD-L1 immunotherapy

First Author*：Nianhong Wu

*** Correspondence:** Pan Li: lipan@hospital.cqmu.edu.cn Supplementary Data

Supplementary Material should be uploaded separately on submission. Please include any supplementary data, figures and/or tables.

# Supplementary Information Text

## Materials and Methods

### *In vitro* Cytotoxicity Assay

To assess the cytotoxicity of MB and LIFU, 4T1 cells (5×10^4^) were seeded in 96-well plates and incubated for 24 hours with MBs concentrations of 10%, 20%, 30% and 40%, or ultrasonic power conditions of 1, 2, 3 and 4 W/cm^2^ (1.0 MHz, 50% duty cycle, 1 minute) for 24 hours before measuring the cell viabilities via the standard CCK-8 assay. The same procedure was used to assess the cytotoxicity profiles against human umbilical vein endothelial cells (HUVECs). In addition, we evaluated the cytotoxicity of LIFU-TMD treatment (MBs concentrations of 10%, 20%, 30% and 40%; 2 W/cm^2^, 1 minute) on 4T1 cells by the standard CCK-8 assay.

### LIFU-TMD treatment for ICD activation *in vitro*

Microbubbles were added to the confocal culture dishes after 4T1 cells had been inoculated, followed by ultrasound irradiation (2 W/cm^2^) for 1 minute. The cells were then fixed with 4% paraformaldehyde after being rinsed with PBS three times. A 5% Bovine Serum Albumin solution (BOSTER) was used to block the cells before incubation with anti-CRT (4°C, overnight; Immunoway). Subsequently, FITC-labeled goat anti-rabbit IgG antibody (Abbkine) was added and incubated at room temperature for 1 hour. The cells were further stained with DAPI for CLSM imaging. Following LIFU-TMD treatment on 4T1 cells in 12-well plates, the cell supernatants were collected and used with the HMGB1 ELISA kit (Jiangsu Meimian Industrial Co.) to measure the amounts of HMGB1 secretion. Similarly, to detect the release of ATP, pre-inoculated 4T1 cells in 12-well plates were subjected to the same treatment. The manufacturer's instructions for the ATP assay kit (Beyotime) were followed to determine the intracellular residual ATP concentration.

### Biosafety of the MBs *in vivo*

Fifteen healthy Babl/c mice were randomly divided into five groups (control group and groups on day 1, 3, 7 and 14 after microbubbles injection). On day 14, blood samples were collected for hematology analysis and major organs (heart, liver, spleen, lung and kidney) were removed for H&E analysis.

## Results

### LIFU-TMD treatment induces ICD *in vitro*

To study the ICD generation, the DAMPs signals were detected *in vitro*. It was discovered that LIFU-TMD treatment significantly increased the green fluorescence of CRT on the membranes compared to other groups (Figure S8). A 1.47-fold increase in HMGB1 levels in the MB+LIFU group, compared with the control group, indicated its release from the nuclei of dying cells (Figure S9). According to the ATP test kit results, the intracellular ATP levels were considerably decreased in the MB+LIFU group, suggesting that more ATP was released into the culture supernatant (Figure S10).

# Supplementary Figures

## Supplementary Figures


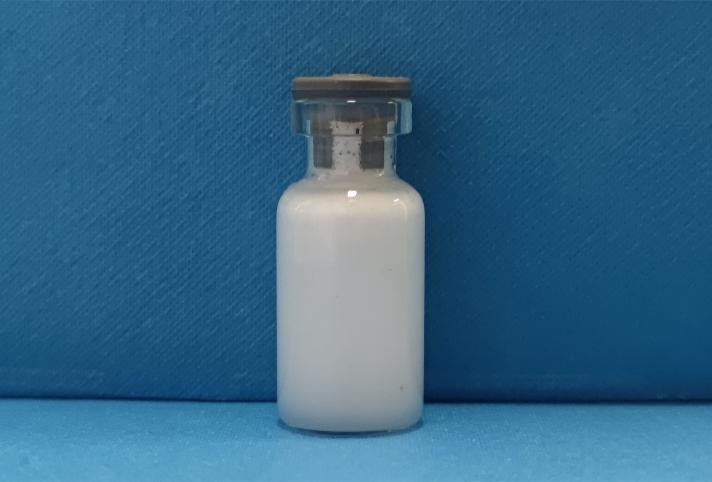


**Supplementary Figure S1.** The physical picture of the MBs.


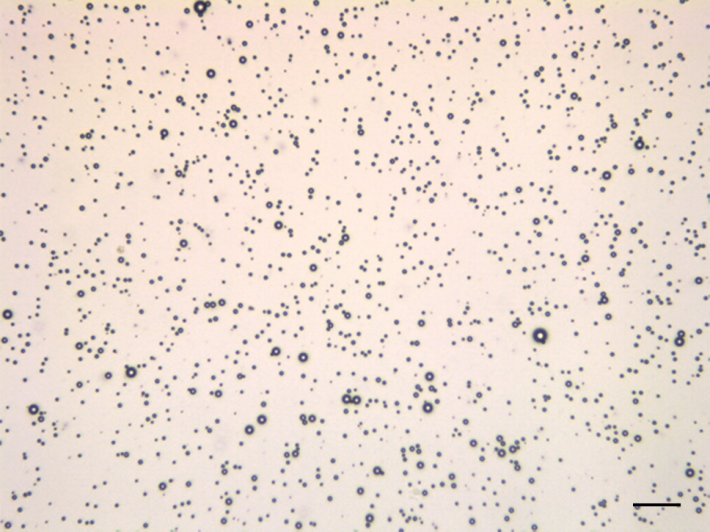


**Supplementary Figure S2.** The optical microscopy image of MBs, the scale bar is 20 μm.


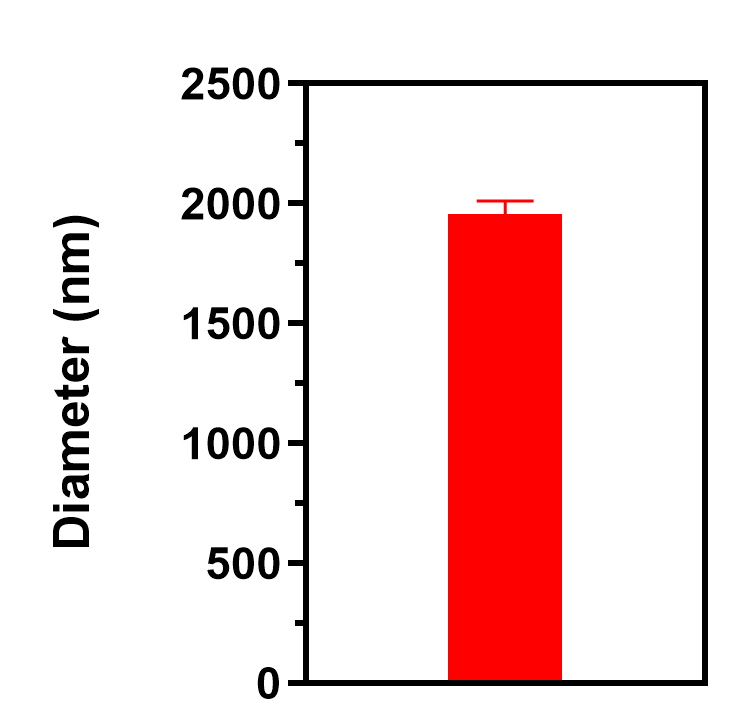


**Supplementary Figure S3.** The average diameter of MBs (n=3).


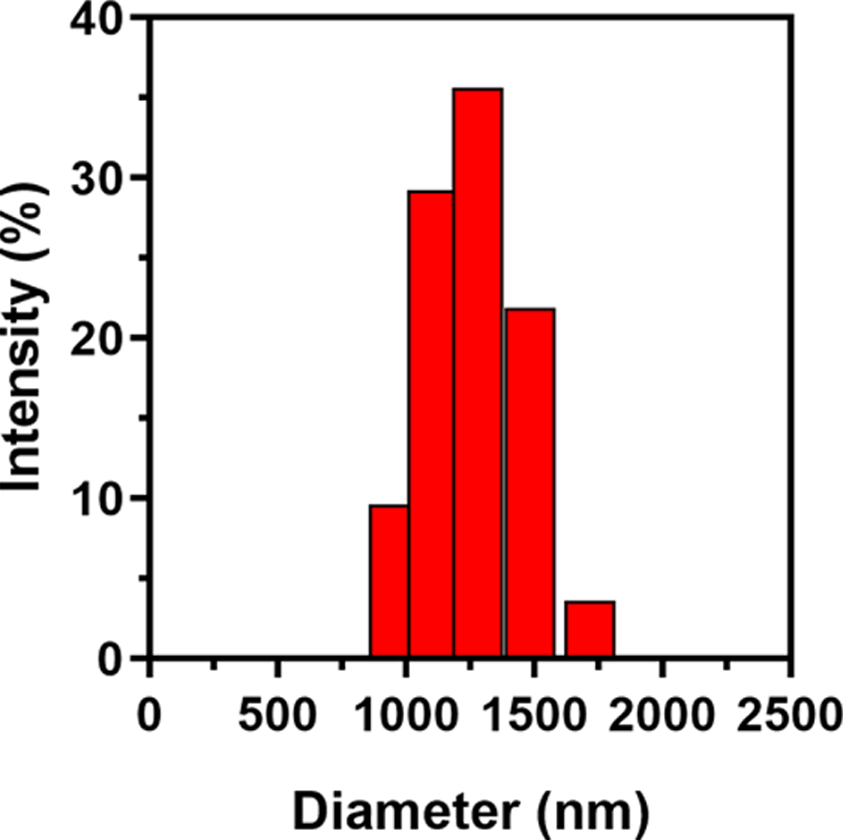


**Supplementary Figure S4.** The size distribution for MBs based on diameter and intensity (%).


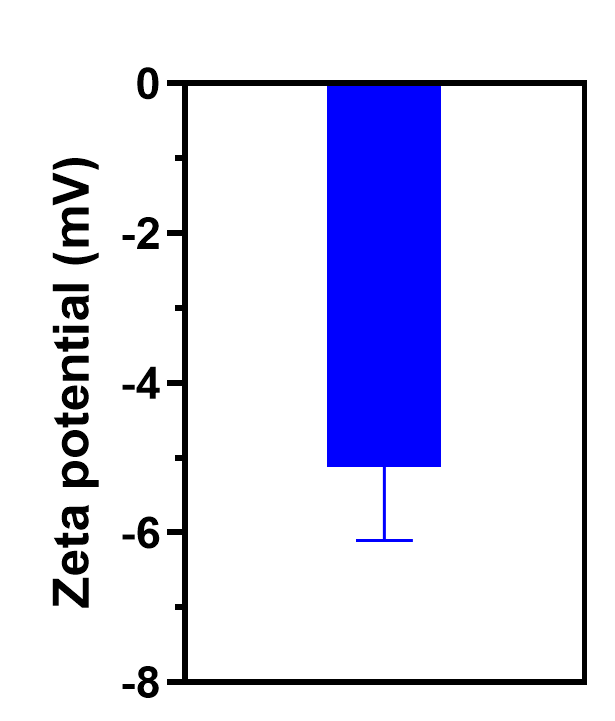


**Supplementary Figure S5.** The zeta potentials of MBs (n=3).


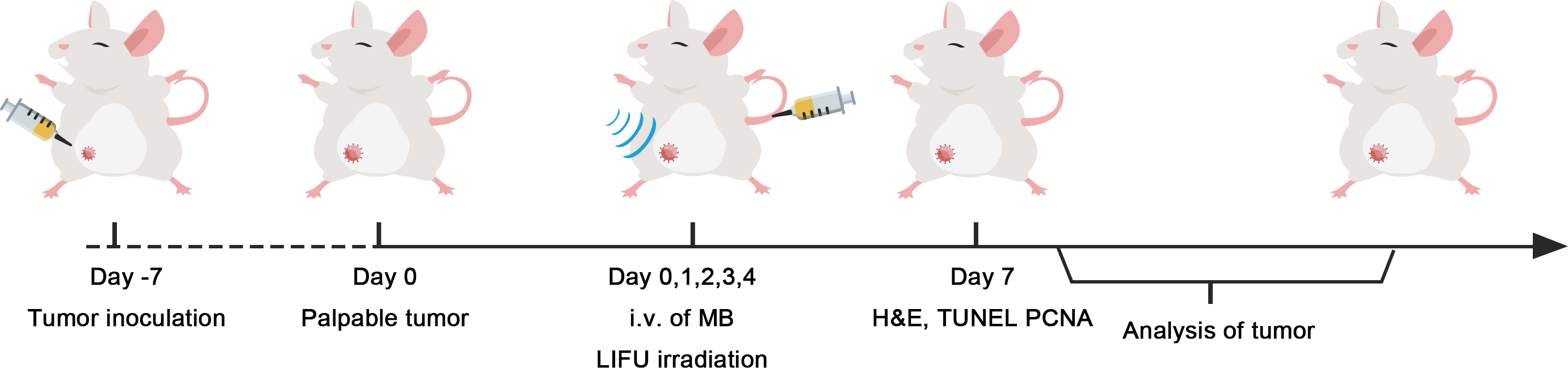


**Supplementary Figure S6.** Schematic illustration of LIFU-MB treatment to suppress the growth of the primary tumors.


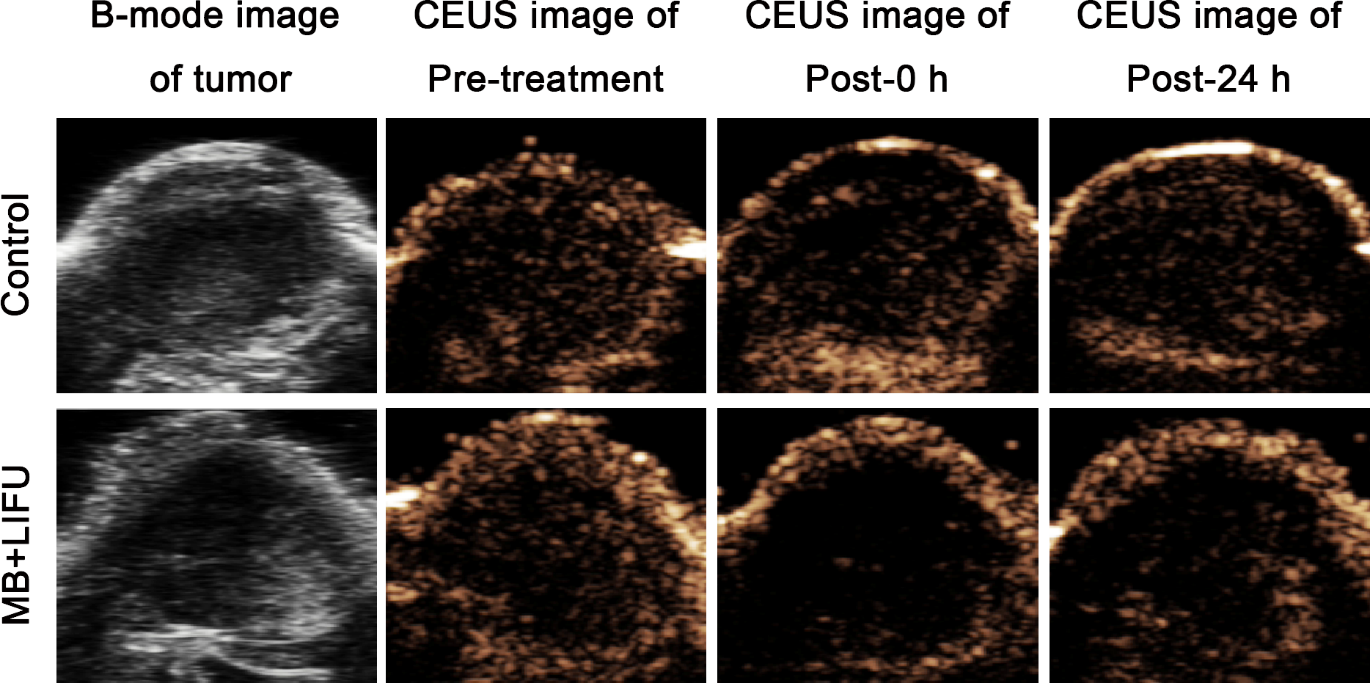


**Supplementary Figure S7.** Representative B-mode and CEUS images of the tumors.


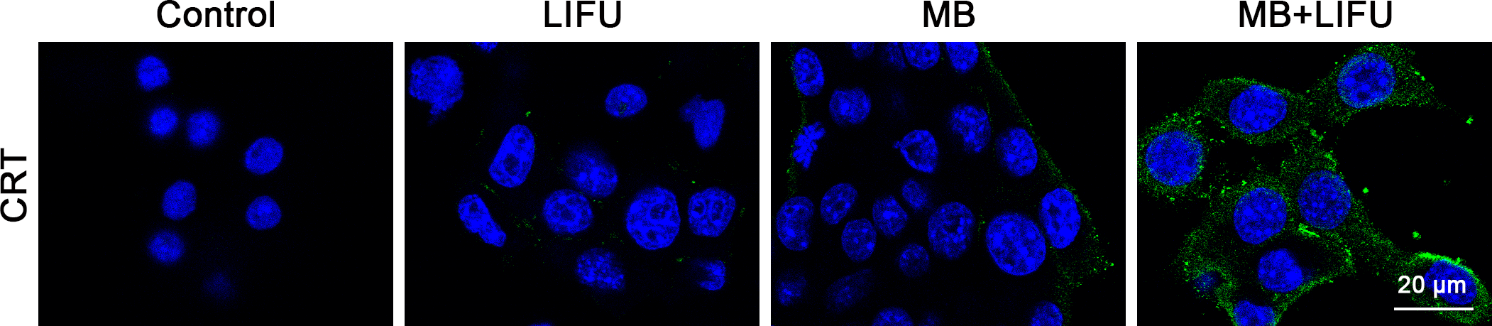


**Supplementary Figure S8.** Confocal microscopic images of 4T1 cells to show CRT exposure after LIFU-TMD treatment. Scale bar is 20 μm.


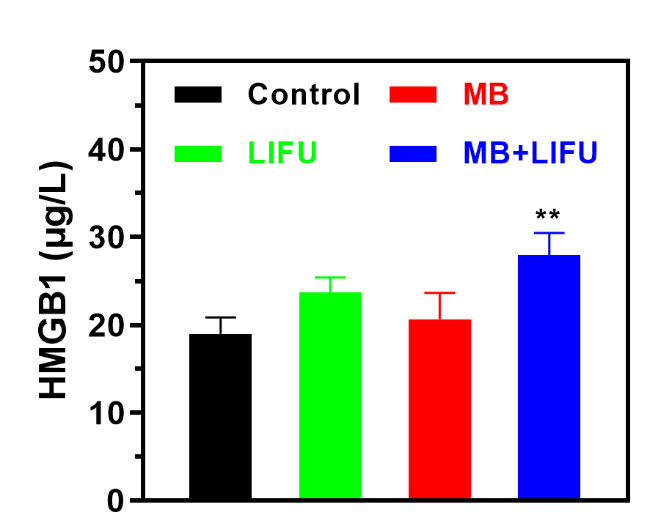


**Supplementary Figure S9.** The release level of HMGB1 after LIFU-TMD treatment (n=3).


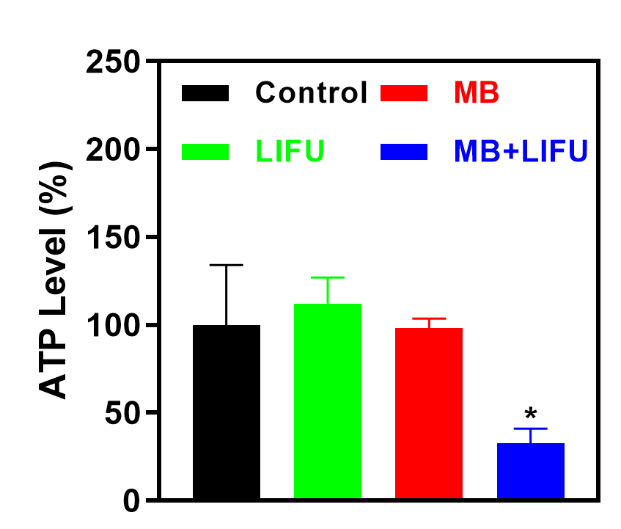


**Supplementary Figure S10.** The intracellular level of ATP after LIFU-TMD treatment (n=3).


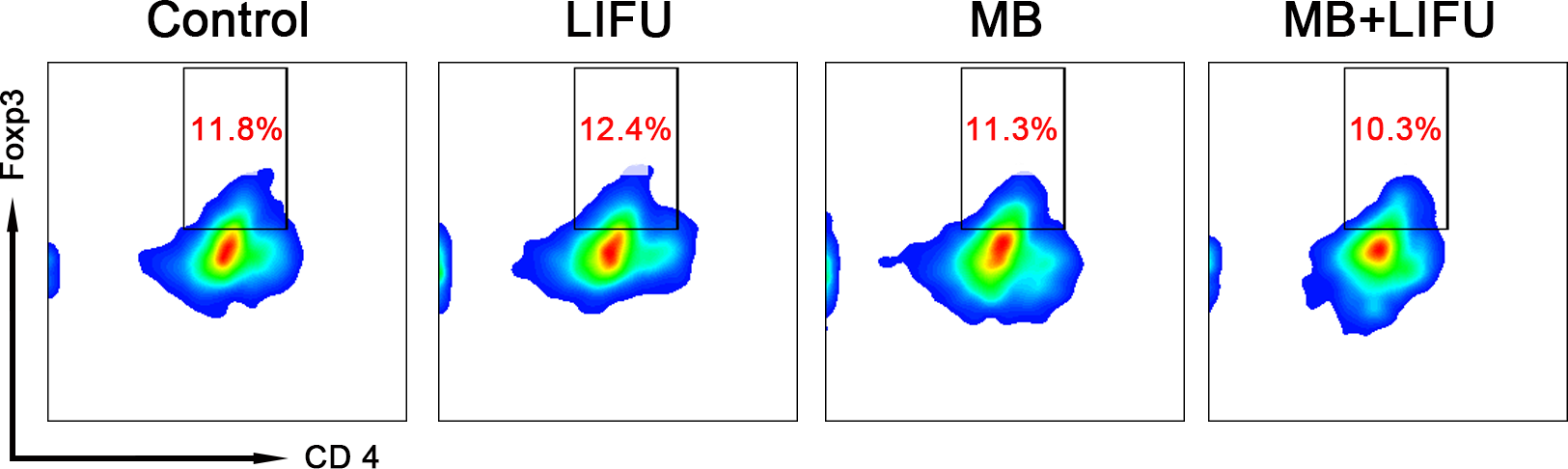


**Supplementary Figure S11.** The flow cytometric analysis of Tregs (CD3^+^CD4^+^Foxp3^+^) in 4T1 tumor-bearing mice.


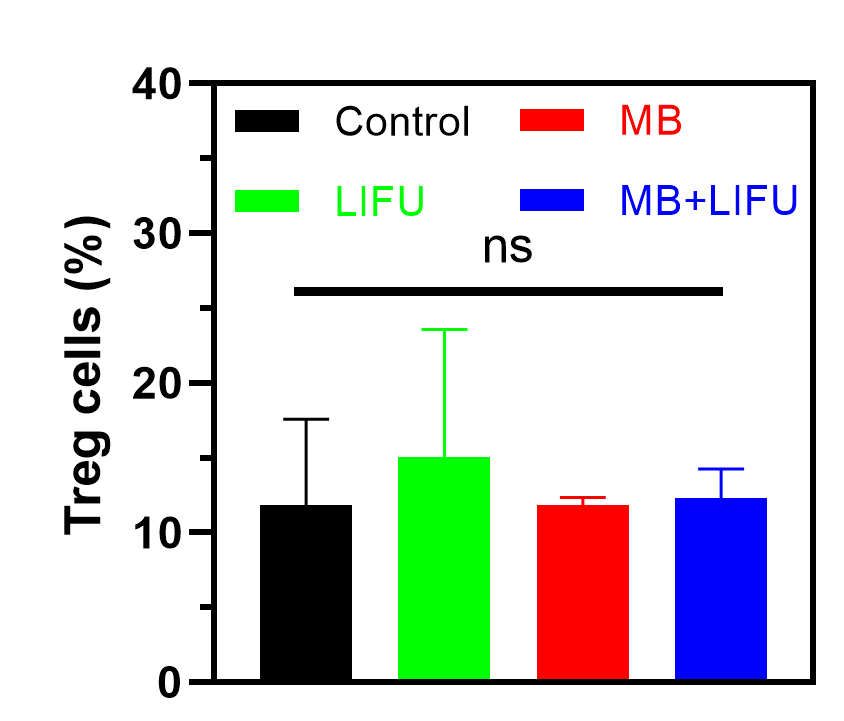


**Supplementary Figure S12.** The corresponding quantification of Tregs (n=3).


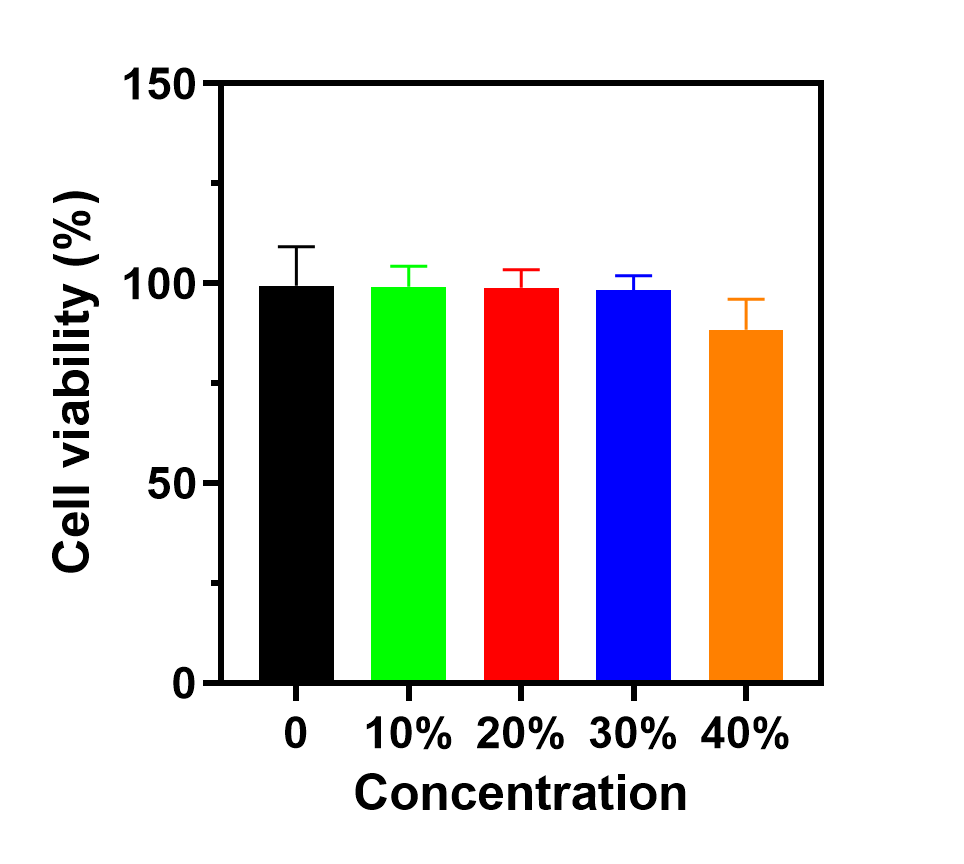


**Supplementary Figure S13.** Relative viability of HUVECs after being treated with MBs at different concentrations for 24 h (n=5).


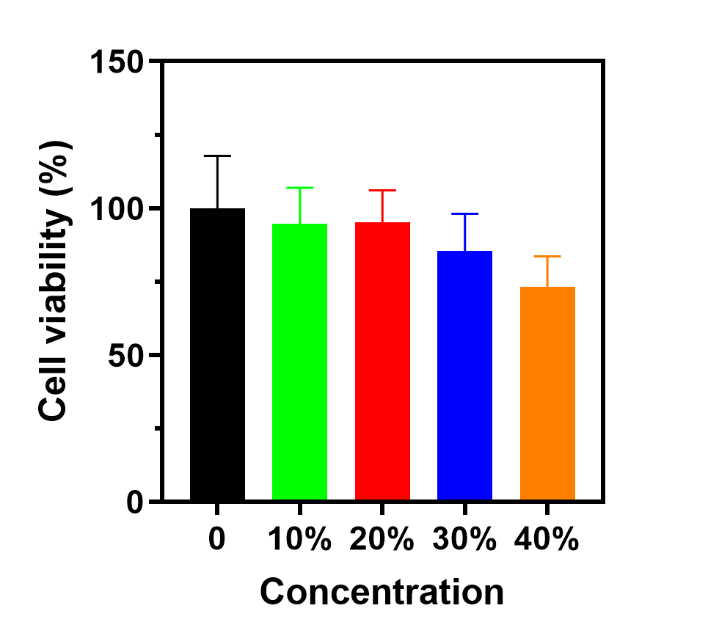


**Supplementary Figure S14.** Relative viability of 4T1 cells after being treated with MBs at different concentrations for 24 h (n=5).


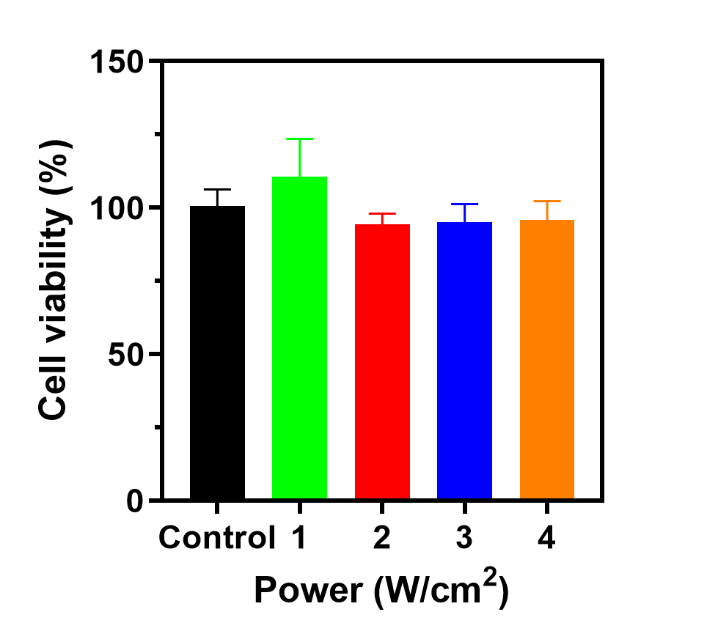


**Supplementary Figure S15.** Relative viability of HUVECs after being treated with LIFU at different power for 24 h (n=5).


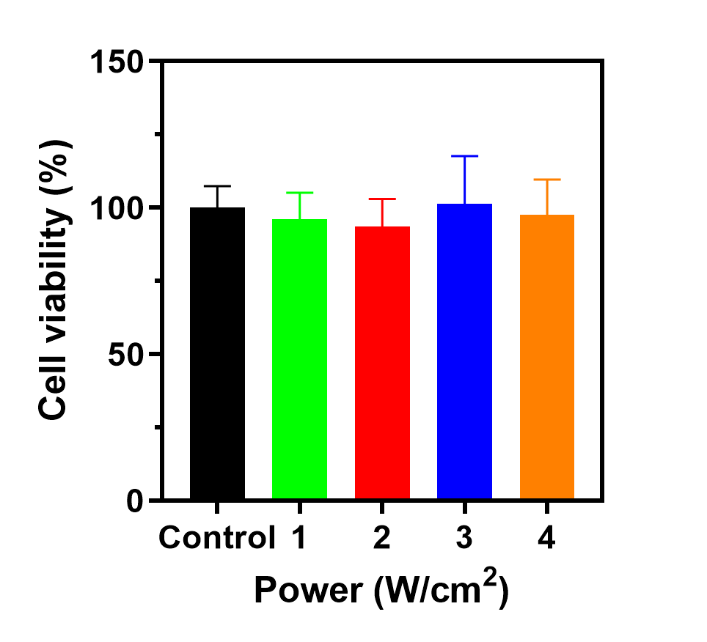


**Supplementary Figure S16.** Relative viability of 4T1 cells after being treated with LIFU at different power for 24 h (n=5).


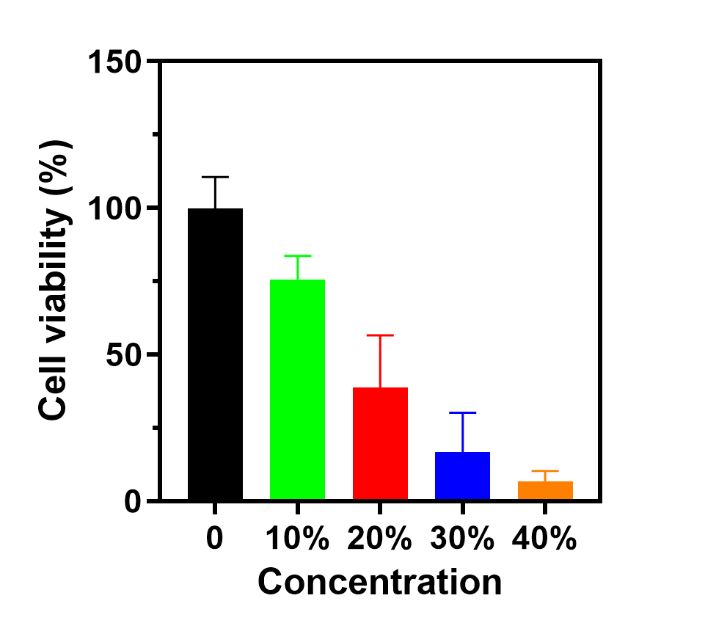


**Supplementary Figure S17.** Relative viability of 4T1 cells after being treated with LIFU with different concentrations of MBs for 24 h (n=5).


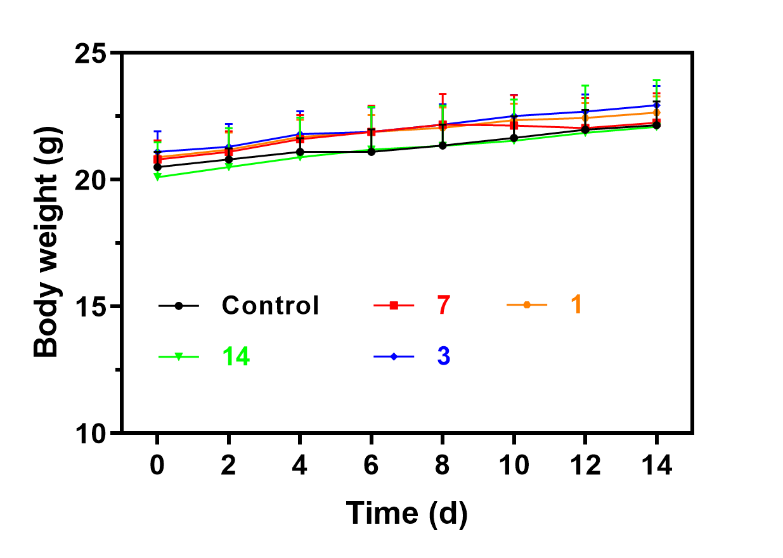


**Supplementary Figure S18.** Body weights changes after MBs injection in tail veins (n=3).
